# A hidden cost of drinking: Alcohol use and gendered inequalities in unpaid care work

**DOI:** 10.1016/j.ssmph.2025.101817

**Published:** 2025-05-16

**Authors:** Teresa Perry

**Affiliations:** California State University, San Bernardino, 5500 University Pkwy, San Bernardino, CA, 92407, USA

## Abstract

The impact of alcohol use on families is an important, yet frequently understudied, avenue of research. Unpaid care work, which encompasses childcare, eldercare, cooking, and cleaning, and alcohol use, are both gendered. Men are more likely to consume alcohol, binge drink, and have alcohol use disorder and women do more unpaid care work in families. Given the gendered nature of both, this study aims to assess if alcohol consumption impacts the distribution of unpaid care work in families by gender. Using the Panel Study of Income Dynamics (PSID), a longitudinal dataset, this paper analyzes how alcohol use influences the husband and wife's distribution of unpaid care work. Data was analyzed using a fixed effects model with robust standard errors. The results indicate that alcohol use significantly impacts the distribution of unpaid care work in the family. Heavy drinking by the husband is associated with the wives completing an additional 10.59 hours of childcare a week and 2.120 more hours of housework a week. Heavy drinking by the wife is correlated with 2.384 fewer hours of housework. The wife's drinking behavior has a minimal effect on the husband's unpaid care burden. These results indicate that there are even higher levels of gender inequality in unpaid care work in alcohol-affected families (AAFs) which may lead to worse social, economic, and psychological outcomes for the women in those families. This further emphasizes the importance of incorporating spouses and families in interventions and policies about alcohol use.

## Highlights

• This study fills a gap in the literature by analyzing the relationship between alcohol use and unpaid care work.
• The results indicate that alcohol consumption enhances existing gender inequalities in unpaid care work.
• When husbands engage in certain drinking behaviors, wives have a larger share of household responsibilities.
• Heavy drinking by husbands is associated with wives taking on 10.59 additional hours of childcare per week.
• Wives' alcohol use had a minimal impact on the amount of unpaid care work performed by husbands.

## 1 Introduction

Globally, around 100 million adult family members are affected by someone else's addiction (Orford et al., 2013). The economic, health, and social consequences for these family members are also profound (Cucciare et al., 2022; McMahon & Giannini, 2003; Sakiyama et al., 2015). Multiple studies have shown that substance abuse in the household contributes to a compromised family environment, where conflict and stress are regularly present within the household (McMahon & Giannini, 2003; National Center for Health Statistics, 1991; Solis et al., 2012). Family members affected by alcohol use disorder are more likely to be diagnosed with depression, substance use disorders, and trauma, as well as to experience higher rates of stress and impaired physical health (Di Sarno et al., 2021; Ray et al., 2009). Their well-being also affects the outcomes for individuals with alcohol use disorders (Timko et al., 2021).

Alcohol-affected families (AAFs) often experience family disruption, where household members may face loss of employment, loss of custody, physical and psychological abuse, and poor health, among other outcomes (Mancheri et al., 2019; Monari et al., 2024; Sharma et al., 2016). This conflict leads to higher rates of household dissolution and divorce for AAFs (Homish et al., 2006). For instance, Caces et al. (1999) found that an increase in alcohol consumption of 1 L per capita was associated with a 20 percent increase in the divorce rate.

The purpose of this paper is to focus on the less-studied impact of alcohol use on unpaid care work. Activities that fall within the category of unpaid care work include caring for the ill, children, and the elderly, as well as cleaning and cooking. Unpaid care work is critical to individual well-being, as people depend on their own or others’ care throughout their lives. Institutional, interpersonal, and social norms influence household decision-making, affecting who does the unpaid care work, who makes financial decisions, and who holds more bargaining power within the household (Agarwal, 1997; Friedberg & Webb, 2006; Miller & Bairoliya, 2022). Research consistently shows that women bear a disproportionate share of the unpaid care work burden, regardless of geographic region, socioeconomic status, or age (Elson, 2017; Ferrant et al., 2014; Martha MacDonald & Lethbridge, 2005; Stanfors et al., 2019). Unpaid care work contributes to broader economic issues, such as the gender wage gap, female labor force participation, and economic inequality by gender, and can lead to worse mental and physical health outcomes for women (Cusatis & Garbarski, 2019; Ferrant et al., 2014; Seedat et al., 2009; Stanfors & Jacobs, 2023).

Alcohol use adds further complexity to these already unequal care dynamics within families. Care is often part of the negotiation process between non-addicted individuals and addicted individuals in the household (Thomas & Ager, 1993). For instance, sober household members often take on additional responsibilities, such as preparing meals for intoxicated family members to help them sober up (Black, 2020). While a partner's alcohol use may cause a sober partner to do more care work, individuals who consume alcohol may also be less likely to contribute to care work in the household. They may engage in destructive behavior or be unable to care for themselves or other household members while intoxicated, increasing the care burden of the other members of the household (Room, 2001). Additionally, even when alcohol-consuming members of the household aren't intoxicated they still might be less likely to do unpaid care work because of the impact that alcohol has on physical and mental health. For example, binge drinkers and frequent high-quantity drinkers have poorer mental health and function, and alcohol use is a significant contributor to illness, disease, and injury, which can decrease the amount of unpaid care work done by people who consume alcohol (Adrian & Barry, 2003; Rehm et al., 2017; Volk et al., 1997).

In the United States there is also a gendered component to alcohol use, as men are more likely than women to binge drink, drink heavily, and develop alcohol use disorders (Keyes et al., 2021; McHugh et al., 2018). Compared to women, men have higher rates of typical drinking frequencies and quantities (Wilsnack et al., 2000). Gender also plays a role in the consequences and outcomes of drinking. For instance, the consequences of heavy alcohol use are worse for women than men, such as being more likely to suffer alcohol-related physical illnesses, more cognitive and motor impairment from alcohol, increased risk of physical harm and sexual assault, and a range of reproductive problems (Nolen-Hoeksema, 2004). While alcohol use by women has enhanced risks, women are also negatively impacted by men's alcohol use. For instance, many studies have linked the use of alcohol by husbands to increased intimate partner violence, increased emotional problems, physical problems, and social problems for wives (Melkam et al., 2024; Sharma et al., 2016). Although this literature has established the gendered consequences of alcohol, little research has examined its broader impact on spousal behavior through a gendered lens. This paper aims to fill that gap.

Because men are more likely to engage in these drinking behaviors and less likely to do unpaid care work, a multiplicative effect of alcohol use on the amount of unpaid care work done by women in AAFs may exist. The multiplicative effect refers to how alcohol use not only increases the overall burden of unpaid care work but also intensifies the pre-existing gender disparity, further deepening the inequities faced by women in these families.

This paper addresses a notable gap in research about the intersection of these two critical issues, unpaid care work and alcohol use, exploring how alcohol use in families impacts the unpaid care burden by gender. By examining the intersection of alcohol use and unpaid care work, this paper contributes to a deeper understanding of how family members are affected by alcohol use. The remainder of the paper is organized as follows. Section 2 outlines the empirical specification, data sources, outcome measures, and statistical analysis; Section 3 presents the results; Section 4 provides a discussion; and Section 5 concludes the paper.

## 2 Methods

### 2.1 Data sources and respondents

The Panel Study of Income Dynamics (PSID) data is utilized to analyze the impact of alcohol use on unpaid care work. PSID is a longitudinal survey that has been tracking various trends among U.S. families since 1968 and tracks the same families over time (Panel Study of Income Dynamics, 2024). Detailed questions about alcohol use were not included until the 2005 wave; therefore, I focus on data from 2005 through 2019. The 2021 survey is excluded due to potential changes in unpaid care work related to the COVID-19 pandemic.

During my sample period for families with two spouses, the husband is coded as the head of household 99.6 percent of the time, and I omit the other 0.4 percent of families for possible bias due to inconsistencies in naming the head of household. However, the respondents to the survey are not always the head of the household, creating another potential bias. Depending on whether the husband or wife was the respondent, the questions regarding the other spouse's allocation of care work and alcohol use are a proxy report. Previous research examines the accuracy of proxy reports and self-reports regarding alcohol use and unpaid care work. For instance, proxy reports about alcohol use are consistent with self-reporting and are unlikely to produce biased results at the aggregate level (Donovan et al., 2004; Graham & Jackson, 1993). Even when self-reported measures of alcohol use are under-reported, for the most part, this information is still reliable and valid (Del Boca & Darkes, 2003; Stockwell et al., 2016). The literature on survey reports about unpaid care work is mixed. Some research suggests that husbands tend to overreport their wives' contributions to unpaid care work, while other studies indicate that both husbands and wives may overreport their respective shares of unpaid care work (Kamo, 2000; Rodrigues et al., 2024; Schröder & Schmiedeberg, 2020).

Due to the mixed results in the literature and to confirm consistent and unbiased estimations across respondents, I disaggregate the regression run in Table 3 by respondent type in Table A1. The magnitude of the results differs based on the respondent, but the majority of the statistically significant results do not change substantially. For instance, the primary results from Table 3 and Table A1 highlight that alcohol use and heavy drinking by the husband result in a statistically significant increase in housework for the wife, regardless of whether the husband or wife is the respondent. However, heavy alcohol use by the wife is statistically significant in the main regression, but not statistically significant for either respondent type when the regression is disaggregated in Table A1. The number of drinks consumed by the husband is negatively associated with housework for the wife, but it is only statistically significant when the husband is the respondent in Table A1. The findings in Table A1, along with the large number of respondents and the longitudinal nature of the data, also help to overcome concerns about reporting biases.

This paper only analyzes the differences in unpaid care work between spouses in AAF and non-AAF families. Thus, single-parent families are not used in the comparison group. Additionally, this paper focuses solely on the alcohol use of the two adults in the household, though future research should investigate families where children consume alcohol. The distinction between how a family and a household is defined varies substantially based on the research area and discipline. PSID defines a household as a specific physical housing unit, and the various families inside that unit are defined as families. This family notation is used for the remainder of the paper.

#### 2.1.1 Outcome measures

From 2005 to 2019 there is only one question about unpaid care work which asks about the amount of housework done by the husband or wife in a typical week. Housework is a continuous variable defined as time spent cooking, cleaning, and doing other tasks around the house in an average week. The question is separated by the amount of housework done by the husband and wife. This is the outcome variable of interest in Table 3.

In 2017 and 2019, the PSID introduced time-use measures to capture information about how individuals allocate their time across various activities. It is important to note that these estimations often miss the role of supervisory roles or multi-tasking and may be an underestimation of the true amount of unpaid care work done in the household. The new data included questions about time spent on personal care, shopping, caring for children, caring for an adult, volunteering, educational activities, and leisure activities during a typical week (Insolera et al., 2019). These variables are continuous variables that measure the average number of hours per week that a person spends doing the specified activity. Table 4 presents the results for these variables for the wife and Table 5 presents the results of these variables for the husband.

#### 2.1.2 Individual measures of alcohol use

**Alcohol Use**: “Does he/she ever drink any alcoholic beverages such as beer, wine, or liquor?”

Two binary variables are created based on this question and whether the husband or wife answers yes (a value of 1) or no (a value of 0). An interaction term is created for alcohol use where the variable is a 1 if both the husband and wife consume alcoholic beverages.

**Heavy Alcohol Use**: “In the last year, on how many days have you had (IF MALE THEN ‘five’/IF FEMALE THEN ‘four’) or more drinks on one occasion?”

The Substance Abuse and Mental Health Services Administration defines heavy alcohol use as binge drinking on 5 or more days in the past 30 days (National Institute on Alcohol Abuse and Alcoholism (NIAAA), 2024). To model this definition, a binary variable is created that is a 1 if the husband or wife binge drinks on 60 or more days in the year, and a 0 otherwise. An interaction term is also created to represent whether both adults in the household drink heavily.

**Frequency of Alcohol Drinks**: “In the last year, on average, how often did he/she have any alcohol to drink?–Would you say, less than one a month, about once a month, several times a month, about once a week, several times a week, or every day?”

The base category consists of respondents who answered less than once a month and binary variables were created for each of the other categories and based on the frequency that alcohol was consumed. For instance, the binary variable for every day would be a 1 if the respondent answered that they drink every day and a 0 otherwise.

**Number of Drinks**: “In the last year, on the days he/she drank, about how many drinks did she have?”

This is a continuous variable that represents the amount of drinks consumed by either the husband or wife on each drinking occasion. This variable has a maximum value of 25 drinks per day.

#### 2.1.3 Control variables

Several variables were included in the regression analysis to help control for factors that also impact unpaid care work. The number of children and people in the household and the hours worked by both the husband and wife directly impact the amount of unpaid care work in the household and how much time they have to complete care tasks, and were included in the analysis. Bertrand et al. (2015) show that the relative hourly earnings of both the husband and wife impact the distribution of unpaid care work in the household, and these variables were also included, in addition to household income. Previous research shows that the age and education level of spouses can also affect the amount of unpaid care work performed by each member of the household, and these variables were included in the regression analysis (Hess et al., 2020). The analysis also included housing status, as homeowners may have higher maintenance duties than renters. Religion, which influences gender norms, family roles, and social expectations, was also included as a control variable. Health status and smoking status were also included because poor health may impact the amount of care that someone can complete and the amount of care that they need. Mental health variable measures were missing for the year 2005 and excluded from the regression analysis in Table 3. However, variables asking the survey respondent about sadness, nervousness, or if everything was an effort were included in the analysis for Table 4, Table 5.

### 2.2 Statistical analysis

A regression analysis, specifically a fixed effects model, was conducted using STATA to address the research question. Fixed effects models are a subset of mixed models and are well-suited for longitudinal data as they control for unobserved heterogeneity, account for time-invariant characteristics, and produce consistent estimators. This approach allows for a focus on within-household variation over time. A Hausman test was conducted to determine whether a fixed or random effects model would be more appropriate for the data, with results strongly favoring the fixed effects specification.<sup>^1^</sup> The unit of analysis is household i over period t, with data collected every two years. The independent variables of interest were included in one regression because the variation inflation factor test indicated that there was a limited concern for multicollinearity. All analyses include robust standard errors, and marginal effects are reported in the results tables for ease of interpretation.

### 2.3 Summary statistics

Table 1 provides summary statistics for the alcohol consumption variables and the full sample from 2005 to 2019. You can find the summary statistics for the control variables in Table A2.

**Table 1:** Summary statistics: 2005–2019

Variable	Obs	Mean	Std. Dev.	Min	Max
**Alcohol Consumption Variables- Husband**					
Consumes Alcohol	34,065	0.6466		0	1
Heavy Alcohol Use	34,065	0.0180		0	1
Number of Drinks	34,065	1.5126	1.8631	0	25
Alcohol Frequency					
Less than Once a Month	34,065	0.1148393		0	1
Once a Month	34,065	0.1058		0	1
Several Times a Month	34,065	0.0920		0	1
Once a Week	34,065	0.1349		0	1
Several Times a Week	34,065	0.1368		0	1
Every Day	34,065	0.0623		0	1
**Alcohol Consumption Variables- Wife**					
Consumes Alcohol	34,065	0.5358		0	1
Heavy Alcohol Use	34,065	0.0052		0	1
Number of Drinks	34,065	0.9520	1.2341	0	25
Alcohol Frequency					
Less than Once a Month	34,065	0.136445		0	1
Once a Month	34,065	0.1038		0	1
Several Times a Month	34,065	0.0808		0	1
Once a Week	34,065	0.0945		0	1
Several Times a Week	34,065	0.0904		0	1
Every Day	34,065	0.0299		0	1
**Interaction Terms**					
Both Consume Alcohol	34,065	0.4679		0	1
Both Binge Drink	34,065	0.0021		0	1
**Panel Data Information**					
Number of Families	5,741				
Average Number of Time Periods	5.934				

Husbands have higher rates of drinking, heavy drinking, and alcohol frequency for all variables. The results also indicate that 64.7 percent of husbands consume alcohol but only 1.8 percent of them drink heavily. Alternatively, 53.6 percent of wives consume alcohol and only 0.5 percent of them drink heavily. Alcohol frequency varies, with husbands being more likely to drink once a week or several times a week whereas wives are more likely to consume alcohol once a month. The percentage of families where both spouses heavily drink is low at 0.2 percent.

## 3 Results

First, I examine how the distribution of unpaid care work between husbands and wives varies based on their alcohol consumption patterns. You can find the corresponding results for the control variables in appendix Table A3, Table A4, Table A5 for all three tables. Table 2 shows the mean values for each type of unpaid care work based on two of the alcohol consumption variables.

**Table 2:** Mean values of spouse variables by drinking categories.

	Personal Care	Shopping	Volunteering	Education	Leisure	Adultcare	Childcare	Housework
Husband's Unpaid Care Work								
*Husband Drinks*								
No	6.956	2.672	1.14	0.686	17.668	1.744	7.602	7.817
Yes	6.571	2.522	1.127	0.711	18.353	1.061	8.204	8.026
*Wife Drinks*								
No	6.901	2.733	1.146	0.74	16.761	2.083	8.241	8.029
Yes	6.543	2.432	1.123	0.664	19.222	0.627	7.748	7.868
*Both Spouses Drink*								
No	6.956	2.694	1.133	0.723	17.306	1.921	8.042	7.93
Yes	6.423	2.433	1.136	0.673	18.992	0.591	7.918	7.964
*Heavy Drinking- Husband*								
No	6.72	2.59	1.153	0.717	17.933	1.31	7.937	7.98
Yes	6.896	2.147	0.239	0.098	26.787	0.555	9.537	6.997
*Heavy Drinking -Wife*								
No	6.716	2.571	1.114	0.704	18.085	1.313	7.953	7.942
Yes	5.714	2.69	0.238	0.119	21.024	0.19	8.571	8.036
*Both Heavily Drink*								
No	6.724	2.579	1.116	0.706	18.106	1.299	7.928	7.947
Yes	4.864	2.773	0.091	0	18.182	0.182	15	8.436

**Wife's Unpaid Care Work**								
*Husband Drinks*								
No	9.728	5.74	1.599	1.051	15.701	5.145	15.446	18.134
Yes	9.476	5.535	1.661	1.227	16.66	2.062	16.627	17.251
*Wife Drinks*								
No	9.71	5.662	1.715	1.102	15.332	4.261	17.683	18.857
Yes	9.461	5.571	1.589	1.215	17.184	2.23	14.934	16.42
*Both Spouses Drink*								
No	9.74	5.693	1.674	1.102	15.664	4.321	17.026	18.486
Yes	9.386	5.524	1.603	1.233	17.077	1.854	15.266	16.502
*Heavy Drink- Husband*								
No	9.536	5.606	1.666	1.174	16.239	3.232	16.021	17.501
Yes	11.055	6.226	1.086	0.834	20.915	1.89	23.366	20.216
*Heavy Drinking- Wife*								
No	9.546	5.599	1.655	1.163	16.264	3.223	16.177	17.578
Yes	10.048	5.833	0.024	1.024	19.857	2.357	14	13.954
*Both Heavily Drink*								
No	9.542	5.607	1.66	1.167	16.283	3.222	16.117	17.555
Yes	10.364	5.545	0.045	1.619	19.045	1.5	24.818	14.808

Consistent with the previous literature, the wife spends more time on all care activities except leisure and volunteering regardless of alcohol consumption behavior. Similarly, wives take on more childcare responsibilities, even when drinking behavior varies, indicating a consistent inequity in the distribution of unpaid care work between husbands and wives.

In general, husbands' and wives’ unpaid care work varies slightly depending on whether both consume alcohol, only one consumes alcohol, or neither consumes alcohol. Consuming alcohol has a minimal impact on most unpaid care activities, with slight variations in the time allocated to leisure, childcare, and housework depending on whether the wife, husband, or both drink. On average, the husband and wife spend more time in leisure when either one of them or both of them drinks.

The effects of heavy drinking on unpaid care work are more pronounced. Husbands who engage in heavy drinking allocate significantly more time to leisure activities and childcare than their counterparts who do not drink heavily. When wives drink heavily, husbands also spend more time on leisure and childcare, suggesting a potential spillover effect. When both partners drink heavily, the husband spends significantly more time on childcare.

A similar pattern exists for wives’ allocation of unpaid care work activities. Wives who heavily drink have significantly more leisure time (19.857 h) compared to non-heavy-drinking wives (16.24 h), while they spend less time on housework, childcare, education, and volunteering. When the husband drinks heavily, the wife also spends more time on housework, personal care, leisure, childcare, and shopping than in families where the husband does not drink heavily. Notably, she dedicates significantly more time to childcare (23.366 h compared to 16.021 h in families without a heavy-drinking husband), on average. When both partners drink heavily, the wife also spends significantly more time on childcare and leisure activities. This suggests that heavy drinking disrupts the usual distribution of unpaid care work, leading to a reduction in productive household contributions and a greater focus on leisure for heavy-drinking spouses.

Table 3 presents the regression results on how the husband and wife's drinking behaviors affect the number of hours of housework done by the husband and wife. Statistically significant coefficients are discussed below.

**Table 3:** Hours of housework 2005–2019 waves in AAFs versus non AAFs.

	Housework- Wife	Housework- Husband
**Alcohol Consumption Variables- Husband**		
Consumes Alcohol	1.097∗∗∗	0.352
	(0.351)	(0.251)
Heavy Alcohol Use	2.120∗∗∗	−0.0124
	(0.746)	(0.439)
Number of Drinks	−0.116∗	0.127∗∗
	(0.0703)	(0.0590)
Alcohol Frequency		
About Once a Month	−0.325	0.0241
	(0.273)	(0.187)
Several Times a Month	−0.545∗	−0.00166
	(0.303)	(0.220)
Once a Week	−0.910∗∗∗	−0.147
	(0.318)	(0.212)
Several Times a Week	−0.394	−0.217
	(0.365)	(0.242)
Every Day	−0.604	0.133
	(0.454)	(0.335)
**Alcohol Consumption Variables- Wife**		
Consumes Alcohol	0.323	−0.0610
	(0.377)	(0.274)
Heavy Alcohol Use	−2.384∗∗∗	0.414
	(0.846)	(1.155)
Number of Drinks	−0.156∗	−0.0567
	(0.0798)	(0.0559)
Alcohol Frequency		
About Once a Month	−0.239	0.172
	(0.239)	(0.175)
Several Times a Month	−0.0273	0.0387
	(0.310)	(0.211)
Once a Week	−0.0881	0.0131
	(0.312)	(0.206)
Several Times a Week	0.192	−0.0353
	(0.366)	(0.258)
Every Day	0.0985	−0.141
	(0.495)	(0.332)
**Interaction Terms**		
Both Spouses Drink	−0.255	−0.0721
	(0.377)	(0.276)
Heavy Alcohol Use- Both Spouses	−0.00229	−0.828
	(1.355)	(1.242)

Observations	34,065	34,065
Number of Families	5,741	5,741

The marginal effects of the coefficient estimates from the fixed effects model with robust standard errors are presented in this table. The control variables in the regression include: the health status of the husband, the health status of the wife, the number of people in the household, the wife smokes cigarettes, the husband smokes cigarettes, interaction term between the two, age of the husband and wife, the hours worked by both the husband and wife, religion, housing status, income of the household, the hourly earnings of the husband, and the hourly earnings of the wife. ∗∗∗ *p <* 0.01, ∗∗ *p <* 0.05, ∗ *p <* 0.1.

The only statistically significant variable that affects the husband's amount of housework per week is the number of drinks he consumes. Holding all else constant, consuming one additional drink per week increases the husband's housework by 0.127 h. In contrast, when the wife partakes in heavy drinking it is associated with 2.384 fewer hours of housework per week compared to when she does not. Heavy alcohol use by the husband is correlated with a 2.120-h increase in the wife's housework per week, a statistically significant effect at the 1 percent level. When the husband drinks, the wife performs approximately 1.097 h more of housework per week compared to families where the husband does not drink.

Table 4 shows the effects of the behavior of household alcohol consumption on the hours spent by the wife on specific unpaid care activities, including caring for children, education, caring for adults, personal care, shopping, volunteering, and leisure. The coefficients show the change in the wife's weekly hours for each activity when the husband or wife drinks, along with interaction effects.

**Table 4:** Hours of Specific Unpaid Care Activities for Wives 2017–2019 Waves in AAFs versus non- AAFs.

	Caring for Child	Education	Caring for Adult	Personal Care	Shopping	Volunteering	Leisure
**Alcohol Consumption Variables- Husband**							
Consumes Alcohol	−0.600	−0.0710	0.861	0.418	0.314	−0.0861	0.107
	(2.817)	(0.320)	(0.998)	(0.590)	(0.428)	(0.327)	(0.790)
Heavy Alcohol Use	10.59∗∗	−0.445	−1.118	0.125	0.293	0.734∗∗	0.475
	(4.939)	(0.696)	(1.353)	(0.958)	(1.034)	(0.374)	(1.485)
Number of Drinks	−0.593	0.0811	0.225	0.451∗∗∗	−0.0185	0.0491	−0.0149
	(0.613)	(0.0872)	(0.223)	(0.159)	(0.127)	(0.0894)	(0.202)
Alcohol Frequency							
About Once a Month	0.433	−0.342	−0.381	−0.538	0.139	0.0972	−0.615
	(2.295)	(0.336)	(0.859)	(0.487)	(0.340)	(0.301)	(0.741)
Several Times a Month	−3.282	0.996∗∗	0.138	−1.071∗	0.234	−0.547	−2.264∗∗
	(2.563)	(0.461)	(1.183)	(0.651)	(0.412)	(0.540)	(0.910)
Once a Week	−1.577	0.609	−1.217	−1.657∗∗∗	0.145	−0.418	−2.155∗∗
	(2.765)	(0.381)	(1.191)	(0.613)	(0.419)	(0.362)	(0.882)
Several Times a Week	1.335	0.272	−1.724	−1.587∗∗	0.215	−0.161	−1.027
	(2.912)	(0.458)	(1.558)	(0.784)	(0.440)	(0.417)	(1.014)
Every Day	−1.269	0.316	3.097	−1.100	0.0390	−0.105	−1.854
	(3.933)	(0.524)	(2.824)	(0.884)	(0.683)	(0.514)	(1.386)
**Alcohol Consumption Variables- Wife**							
Consumes Alcohol	1.200	−0.374	3.073∗	0.494	−0.0289	0.0698	−0.659
	(3.448)	(0.591)	(1.715)	(0.651)	(0.537)	(0.289)	(1.172)
Heavy Alcohol Use	1.444	−0.0124	1.914	−0.366	1.576	−0.340	−5.785∗∗∗
	(5.043)	(1.517)	(2.713)	(1.365)	(1.399)	(0.418)	(2.017)
Number of Drinks	0.145	−0.274∗∗	−0.267	−0.0445	−0.0171	0.251	0.116
	(0.786)	(0.120)	(0.200)	(0.171)	(0.113)	(0.232)	(0.298)
Alcohol Frequency							
About Once a Month	−5.455∗∗	0.783∗∗	−0.881	0.542	0.277	0.164	0.490
	(2.173)	(0.324)	(0.800)	(0.409)	(0.314)	(0.248)	(0.724)
Several Times a Month	−6.464∗∗	0.410	0.716	1.043∗∗	0.191	0.242	0.240
	(2.792)	(0.422)	(1.000)	(0.490)	(0.401)	(0.293)	(0.890)
Once a Week	−6.748∗∗	0.465	−0.268	0.841	0.316	0.107	−0.324
	(2.658)	(0.407)	(0.964)	(0.569)	(0.449)	(0.334)	(0.942)
Several Times a Week	−6.712∗∗	0.837∗	0.646	1.365∗	0.249	−0.0396	−1.439
	(2.777)	(0.462)	(1.118)	(0.772)	(0.539)	(0.328)	(1.024)
Every Day	−5.163∗	0.837	−1.297	−0.793	−1.188	0.587	−1.431
	(3.057)	(0.528)	(1.316)	(0.987)	(0.740)	(0.406)	(1.678)
**Interaction Terms**							
Both Spouses Drink	1.919	0.392	−3.803∗∗	−1.391∗∗	0.248	−0.194	1.163
	(3.437)	(0.512)	(1.931)	(0.611)	(0.550)	(0.422)	(1.145)
Heavy Alcohol Use- Both Spouses	−3.737	−0.673	3.398	−0.685	−1.048	−1.103∗	4.883∗
	(9.091)	(1.879)	(3.547)	(2.003)	(2.093)	(0.617)	(2.776)

Observations	8,215	8,233	8,227	8,221	8,218	8,228	8,226

The marginal effects of the coefficient estimates from the fixed effects model with robust standard errors are presented in this table. The control variables in the regression include: the health status of the wife, the health status of the husband, variables measuring if everything felt like an effort, nervousness, and sadness for the reference person, the number of people, the wife smokes cigarettes, husband smokes cigarettes, interaction term between the two, age of husband and wife, the hours worked by both the husband and wife, religion, housing status, income of the household, the hourly earnings of the husband, and the hourly earnings of the wife. ∗∗∗ *p <* 0.01, ∗∗ *p <* 0.05, ∗ *p <* 0.1.

When the husband engages in heavy alcohol consumption, it is linked to the wife spending an additional 10.59 h per week on childcare. This association is statistically significant at the 5 percent level. When the husband drinks once a week, she spends less time on leisure and personal care, and if he drinks several times a month she spends less time on leisure.

On average, heavy alcohol use by the wife is associated with a statistically significant decrease of 5.785 h per week in her leisure time. Drinking about once a month, several times a month, once a week, several times a week, and every day is also correlated with a statistically significant decrease in her time spent caring for children. When both spouses drink there is less time spent on personal care and caring for an adult. Both spouses drinking heavily is correlated with a statistically significant increase in the wife's leisure of 4.883 h per week.

Table 5 shows how much the husband spends on specific unpaid care activities based on variations in the drinking behavior of himself and his wife.

**Table 5:** Hours of Specific Unpaid Care Activities for Husbands 2017–2019 Waves in AAFs versus non-AAFs.

	Caring for Child	Education	Caring for Adult	Personal Care	Shopping	Volunteering	Leisure
**Alcohol Consumption Variables- Husband**							
Consumes Alcohol	−2.246	0.0738	−0.716	0.684∗∗	−0.124	0.254	0.502
	(2.111)	(0.272)	(1.188)	(0.343)	(0.263)	(0.218)	(1.068)
Heavy Alcohol Use 2.573	−0.0550	1.206	0.143	−0.961∗∗∗	0.138	4.719∗∗	
	(2.938)	(0.179)	(0.987)	(0.632)	(0.358)	(0.190)	(2.078)
Number of Drinks	−0.249	0.118∗∗	−0.0858	−0.113	0.0492	−0.0742	0.372
	(0.587)	(0.0525)	(0.0932)	(0.0793)	(0.0691)	(0.0492)	(0.253)
Alcohol Frequency							
About Once a Month	0.850	−0.389	0.119	−0.230	−0.297	−0.0567	−2.552∗∗∗
	(1.967)	(0.259)	(0.661)	(0.320)	(0.212)	(0.201)	(0.988)
Several Times a Month	−0.198	−0.0219	0.487	−1.094∗∗	0.481∗	−0.129	−3.668∗∗∗
	(2.336)	(0.257)	(0.657)	(0.439)	(0.274)	(0.235)	(1.067)
Once a Week	−0.215	−0.317	0.318	−0.839∗∗	0.129	−0.0458	−5.426∗∗∗
	(2.488)	(0.267)	(0.613)	(0.400)	(0.238)	(0.220)	(1.166)
Several Times a Week	0.230	0.0245	1.147	−0.273	−0.110	0.0470	−5.129∗∗∗
	(2.481)	(0.270)	(0.701)	(0.430)	(0.280)	(0.245)	(1.263)
Every Day	−0.958	0.223	0.897	0.832	−0.448	−0.110	−6.111∗∗∗
	(2.682)	(0.335)	(0.689)	(0.652)	(0.375)	(0.294)	(1.907)
**Alcohol Consumption Variables- Wife**							
Consumes Alcohol	−1.878	0.113	−1.097	−0.241	−0.320	0.292	−1.087
	(3.061)	(0.480)	(0.850)	(0.681)	(0.346)	(0.198)	(1.858)
Heavy Alcohol Use	0.475	−0.0991	0.543	4.108∗	1.353	−0.109	−0.677
	(4.708)	(0.794)	(0.548)	(2.470)	(0.915)	(0.173)	(6.410)
Number of Drinks −0.0667	−0.0340	0.0398	0.122	0.117∗	−0.0529	−0.0194	
(0.627)	(0.0698)	(0.0872)	(0.111)	(0.0613)	(0.0599)	(0.367)	
Alcohol Frequency							
Once a Month	−2.794∗	−0.163	0.926∗∗∗	−0.0987	0.129	0.187	2.025∗∗
	(1.661)	(0.215)	(0.315)	(0.305)	(0.187)	(0.169)	(0.864)
Several Times a Month	−2.907	−0.279	0.484	0.679	0.0390	0.161	0.420
	(1.974)	(0.284)	(0.402)	(0.454)	(0.258)	(0.188)	(0.980)
Once a Week	−1.428	−0.0889	0.602∗	0.455	0.636∗∗	0.0780	2.438∗∗
	(2.028)	(0.288)	(0.354)	(0.435)	(0.251)	(0.230)	(1.088)
Several Times a Week	−2.214	−0.110	−0.180	0.0729	0.737∗∗	0.0308	0.404
	(2.137)	(0.372)	(0.435)	(0.500)	(0.308)	(0.281)	(1.267)
Every Day	−0.297	0.0168	0.125	−1.493∗	0.778	0.439	0.473
	(2.150)	(0.354)	(0.434)	(0.873)	(0.526)	(0.271)	(1.789)
**Interaction Terms**							
Both Spouses Drink	1.942	0.290	0.531	−0.345	0.161	−0.271	2.424
	(2.869)	(0.434)	(0.970)	(0.657)	(0.335)	(0.207)	(1.822)
Heavy Alcohol Use- Both Spouses	9.535	−0.148	−1.323	−7.268∗∗∗	0.850	0.0543	−5.851
	(8.283)	(0.761)	(1.085)	(2.613)	(0.971)	(0.293)	(6.780)

Observations	8,216	8,226	8,233	8,210	8,225	8,229	8,215

The marginal effects of the coefficient estimates from the fixed effects model with robust standard errors are presented in this table. The control variables in the regression include: the health status of the wife, the health status of the husband, variables measuring if everything felt like an effort, nervousness, and sadness for the reference person, the number of people, the wife smokes cigarettes, husband smokes cigarettes, interaction term between the two, age of husband and wife, the hours worked by both the husband and wife, religion, housing status, income of the household, the hourly earnings of the husband, and the hourly earnings of the wife. ∗∗∗ *p <* 0.01, ∗∗ *p <* 0.05, ∗ *p <* 0.1.

Heavy drinking by the husband is associated with a statistically significant decrease in shopping time and an increase of 4.719 h of leisure per week. Increases in the husband's frequency of drinking are associated with a decrease in his leisure time which ranges from 2.552 h per week to 6.111 h per week.

Heavy alcohol use by the wife is associated with a 4.108-h increase in the husband's amount of time spent on personal care. Furthermore, there is an association between the frequency of drinking of the wife and the husband's time spent shopping and leisure. The interaction terms indicate that there is a negative correlation between both spouse's heavy drinking and personal care, with there being a 7.268 decrease in hours spent on personal care.

Reverse causality and time-invariant heterogeneity are primary concerns for this study. Using a Monte Carlo simulation, Leszczensky and Wolbring (2022) demonstrate that a cross-lagged panel model with fixed effects, estimated using the maximum likelihood structural equation modeling (ML-SEM) method, can address these issues to some extent. To help address these concerns, I utilize this estimation method and incorporate contemporaneous and two lagged periods of the independent variables of interest and one lag of the dependent variable. The lagged value of the dependent variable serves as an explanatory variable for the independent regressors. The ML-SEM method accounts for reverse causality by assuming sequential exogeneity for the regressors, allowing for the residuals to correlate with the future values of the predetermined explanatory variables (Leszczensky & Wolbring, 2022). Due to the necessary number of lags, the regression can only be replicated in Table 3. The results are in Table A6.

As a second robustness check, and to help address endogeneity concerns, I utilize the Arellano bond estimator, a type of Generalized Method of Moments (GMM) model, and the results are presented in Table A7. This model addresses issues of endogeneity, autocorrelation, and unobserved heterogeneity (Ullah et al., 2018). Lags of the dependent variables are included as explanatory variables in the regressions, and their first and second lags are included as instruments. The independent variables of interest (i.e. the alcohol-related variables) are also instrumented using their first lagged values. Including the lagged values as indicators helps to mitigate endogeneity caused by correlations with unobserved fixed effects and ensures that the variables are not biased by omitted variables. Similarly to Table A6, due to the necessary lags, the results can only be replicated for Table 3.

All three models vary significantly in their estimation methods, meaning that there will be differences in the coefficient estimates. To ensure that reverse causality and endogeneity are not driving the results in Table 3 and I examine the statistical significance and direction of the results rather than the magnitude of the coefficient estimates. Although the magnitude of the results varies in Table A6, heavy alcohol use and alcohol consumption by the husband has a positive association with the housework of the wife and are statistically significant. The wife's heavy alcohol use still has a negative correlation with the amount of housework but is not statistically significant in Table A6. Heavy alcohol use by the wife has a negative correlation with her housework amount and is also statistically significant in Table A7. However, the husband's consumption of alcohol has a negative, but statistically insignificant sign. Table A6, Table A7 indicate that heavy alcohol use by the husband has a positive association with the housework of the wife and is statistically significant. Both robustness checks give further confidence that the results in the paper are not driven by reverse causality or endogeneity.

## 4 Discussion

The results highlight that alcohol consumption has an impact on the allocation of unpaid care work completed by both husbands and wives. Heavier drinking is associated with less time spent on productive household activities and enhances the gender disparity in household responsibilities, with women consistently performing more unpaid care work. A particularly concerning finding is that heavy alcohol use by the husband is associated with 10.59 more hours of childcare per week for wives when their husbands drink heavily. Over a 30-day month, this translates to approximately 45.39 additional hours of childcare, on top of the already disproportionate childcare burden that women typically bear within families. Wives may be taking on more unpaid care work in an attempt to mitigate the adverse impact of their husband's drinking on their children. For instance, the children of people with alcohol and substance use disorders often experience higher levels of abuse and neglect, economic hardship, social isolation, and worse mental and physical health (Grant, 2000; Turney & Olsen, 2019).

While wives who heavily drink spend more time on leisure, on average, once I add control variables to the regression, heavy drinking by wives, is associated with a decrease in their leisure time and has no statistically significant effect on the husband's unpaid care work. This suggests that women may be sacrificing leisure time to consume alcohol, rather than reducing time spent on childcare or other care activities. The results also indicate that heavy drinking by the husband results in a reallocation of his time away from productive household activities and toward leisure or adult care responsibilities, potentially reducing his engagement in routine household tasks such as shopping and volunteering. Moderate alcohol consumption by wives, on the other hand, may be linked to a more balanced division of household responsibilities, with husbands taking on a greater share of flexible tasks.

The wife's alcohol consumption has a minimal effect on the amount of unpaid care work performed by her husband, reflecting gender norms around caregiving. The fact that wives perform significantly more childcare and housework when their husbands engage in heavy drinking, while the husband's care contributions remain relatively unchanged when wives consume alcohol, highlights the entrenchment of traditional gender norms around caregiving. The results also suggest a multiplicative effect of alcohol consumption on women's unpaid care work. Women in these families take on more childcare and more housework than those in families without heavy drinking. This dynamic suggests that alcohol use may enhance pre-existing gender inequalities in household labor, further intensifying the “double burden” faced by women in these contexts. Thus, the additional unpaid care work performed by wives in AAFs not only reflects a coping strategy for managing the negative consequences of a partner's drinking but also perpetuates a broader pattern of gendered inequity in household labor distribution. A larger unpaid care work burden for wives results in worse outcomes for them across multiple fronts, including mental and physical health, economic and career well-being, and social welfare (Ferrant et al., 2014; Jung & O’Brien, 2019).

The gendered patterns observed in the distribution of unpaid care work in AAFs may further strain family relationships, as wives may feel resentment or frustration over the lack of support from their partners. This may be a contributing factor to why AAFs experience high rates of conflict and household dissolution. These results also have important implications for addiction treatment and family intervention strategies. Most interventions focus on the individual who uses alcohol, but the affected family members are also impacted by alcohol use. However, in line with previous research, treatment has benefits for affected family members (see for instance Shanahan et al. (2020)) and these benefits need to be considered in the addiction treatment system.

### 4.1 Limitations

This study has several limitations. First, while the two robustness checks coupled with the fixed-effects model help address endogeneity concerns, the results still need to be interpreted cautiously. Secondly, this paper only analyzes the amount of unpaid care work completed by the husband and wife and does not analyze the impact of alcohol use on the unpaid care work done by other household members or children. This is an important extension for future research. The variation in proxy reports on unpaid care work is another important consideration when analyzing the results. The specification of the alcohol consumption variables might be missing important nuances in problematic drinking that may influence household labor. This paper also limits itself to married heterosexual couples, excluding other less traditional family structures, such as single-parent or multigenerational families. In future research, it is necessary to study the effect of alcohol use on these types of families and its impact on unpaid care work for children.

The data only includes families in the United States, but it is important to understand if these results would be relevant in other countries. By gender, North America has the most even unpaid care work distribution of any other region in the world (Ferrant et al., 2014). Across all countries men are more likely to partake in most drinking behaviors, meaning that the multiplicative effect is likely stronger in some countries (Chaiyasong et al., 2018). This is another important avenue of future research.

## 5 Conclusion

This study illustrates that alcohol use significantly impacts the distribution of unpaid care work within families in the United States. Heavy drinking is a driving factor that leads to gender disparities in unpaid care activities. Wives bear a significantly larger share of the childcare and household responsibilities when husbands partake in heavy drinking, while husbands do not. This enhances existing gender inequalities in unpaid care work, creating a multiplicative effect on the burden of women in AAFs. This multiplicative effect indicates that women in these families may be negatively impacted through economic, social, psychological, and physical avenues. Addiction research and intervention strategies must consider, not only the individual struggling with alcohol use, but also the family members who bear the hidden costs of that use.

## Ethical statement

All ethical guidelines have been followed.

## Financial disclosure

Note to declare.

## Declaration of competing interest

The authors declare that they have no known competing financial interests or personal relationships that could have appeared to influence the work reported in this paper.

## Figures and Tables

**Table A1:** Hours of Housework 2005–2019 AAFs versus NonAAFs Disagraggregated by Respondent Type

	Husband is Respondent		Wife is Respondent	
	Housework- Wife	Housework- Husband	Housework- Wife	Housework- Husband
**Alcohol Consumption Variables- Husband**				
Consumes Alcohol	1.281∗∗	0.639	1.476∗∗∗	0.573∗
	(0.498)	(0.409)	(0.489)	(0.295)
Heavy Alcohol Use	2.936∗∗	−0.616	2.084∗∗	0.360
	(1.184)	(0.617)	(1.004)	(0.503)
Number of Drinks	−0.160∗	0.162	−0.0742	0.0832∗∗
	(0.0892)	(0.108)	(0.105)	(0.0388)
Alcohol Frequency				
About Once a Month	−0.312	−0.451	−0.612∗	0.103
	(0.435)	(0.304)	(0.357)	(0.226)
Several Times a Month	−1.294∗∗∗	0.124	−0.213	−0.487∗
	(0.477)	(0.386)	(0.409)	(0.280)
Once a Week	−1.678∗∗∗	−0.293	−0.485	−0.357
	(0.487)	(0.366)	(0.432)	(0.269)
Several Times a Week	−1.110∗∗	−0.686	−0.00594	−0.262
	(0.525)	(0.435)	(0.523)	(0.321)
Every Day	−0.873	0.599	−0.542	−0.415
	(0.679)	(0.566)	(0.632)	(0.453)
**Alcohol Consumption Variables- Wife**				
Consumes Alcohol	−0.801	−0.153	1.298∗∗	0.355
	(0.570)	(0.458)	(0.507)	(0.322)
Heavy Alcohol Use	−1.390	0.143	−0.126	1.268
	(0.895)	(0.828)	(1.808)	(2.556)
Number of Drinks	0.00885	0.0561	−0.207∗	−0.170∗∗
	(0.129)	(0.0980)	(0.107)	(0.0674)
Alcohol Frequency				
About Once a Month	0.309	0.116	−0.503∗	0.144
	(0.409)	(0.283)	(0.304)	(0.232)
Several Times a Month	0.862∗	−0.174	−0.487	0.161
	(0.479)	(0.320)	(0.418)	(0.267)
Once a Week	1.349∗∗∗	0.00427	−0.869∗∗	0.174
	(0.480)	(0.325)	(0.418)	(0.277)
Several Times a Week	1.304∗∗∗	0.380	−0.519	−0.314
	(0.506)	(0.375)	(0.559)	(0.394)
Every Day	1.302∗∗	0.141	−1.326	−0.530
	(0.615)	(0.431)	(0.822)	(0.503)
**Interaction Terms**				
Both Spouses Drink	−0.0553	−0.262	−0.681	0.00707
	(0.529)	(0.445)	(0.510)	(0.335)
Heavy Alcohol Use- Both Spouses	−1.769	−0.609	−1.099	−1.075
	(2.002)	(1.209)	(2.186)	(2.355)

Observations	14,466	14,749	18,349	18,487

The table presents the estimated marginal effects from the fixed effects model with robust standard errors in parentheses. ∗∗∗.

*p <* 0.01, ∗∗ *p <* 0.05, ∗ *p <* 0.1.

**Table A2:** Descriptive Statistics- Control Variables

Variable	Obs	Mean	Std. Dev.	Min	Max
Number of People	34,065	3.2555	1.3829	2	11
Health Status- Wife					
Very Good	34,065	0.3673		0	1
Good	34,065	0.3088		0	1
Fair	34,065	0.1069		0	1
Poor	34,065	0.0313		0	1
Health Status- Head					
Very Good	34,065	0.3583		0	1
Good	34,065	0.3338		0	1
Fair	34,065	0.1156		0	1
Poor	34,065	0.0293		0	1
Both Smoke	34,065	0.0650		0	1
Husband Currently Smokes	34,065	0.1256		0	1
Wife Currently Smokes	34,065	0.1600		0	1
Age- Husband	34,065	51.4137	13.4193	18	95
Age- Wife	34,065	49.4914	17.3977	17	91
Number of Children	34,065	0.8635	1.1970	0	9
Received College Degree- Spouse					
Yes	34,065	0.3821		0	1
No	34,065	0.1915		0	1
Received College Degree- Husband					
Yes	34,065	0.3621		0	1
No	34,065	0.1767		0	1
Annual Hours on Job- Wife	34,065	1217.165	969.2194	0	5200
Annual Hours on Job- Husband	34,065	1731.729	1054.853	0	5824
Housing Status					
Rents	34,065	0.1731		0	1
Neither Owns nor Rents	34,065	0.0128		0	1
Income	34,065	16360.63	383364.2	0	3800000
Wage Rate- Husband	34,065	25.0222	37.7059	0	960.65
Wage Rate- Wife	34,065	15.6226	24.9153	0	969.04

**Table A3:** Hours of Housework 2005–2019 Waves in AAFs versus Non AAFs- Control Variable Re- sults

	Housework- Wife	Housework- Husband
Number of People	0.725∗∗∗	0.0343
	(0.139)	(0.0899)
Health Status- Head		
Very Good	−0.0969	−0.195
	(0.215)	(0.139)
Good	−0.166	−0.699∗∗∗
	(0.263)	(0.173)
Fair	−0.224	−1.049∗∗∗
	(0.412)	(0.248)
Poor	0.146	−2.495∗∗∗
	(0.662)	(0.412)
Health Status- Wife Very Good	0.0528	−0.176
	(0.230)	(0.142)
Good	−0.0955	0.239
	(0.283)	(0.173)
Fair	−1.475∗∗∗	0.413
	(0.383)	(0.253)
Poor	−3.674∗∗∗	2.049∗∗∗
	(0.636)	(0.496)
Both Smoke	−1.492∗∗	0.223
	(0.713)	(0.431)
Wife Currently Smokes	0.826∗	0.208
	(0.453)	(0.273)
Husband Currently Smokes	0.681∗	0.316
	(0.388)	(0.278)
Age- Husband	−0.00254	−0.00719
	(0.0166)	(0.0117)
Age- Wife	0.0111∗∗∗	0.00668
	(0.00348)	(0.00556)
Number of Children	0.739∗∗∗	0.326∗∗∗
	(0.139)	(0.0975)
Received College Degree- Wife		
Yes	−0.796∗	0.0294
	(0.407)	(0.286)
No	−0.846∗∗	0.324
	(0.356)	(0.243)
Received College Degree- Husband		
Yes	−0.554	−0.282
	(0.442)	(0.287)
No	−0.687∗	−0.349
	(0.357)	(0.267)
Annual Hours on Job- Wife	−0.00300∗∗∗	0.000677∗∗∗
	(0.000119)	(7.59e-05)
Annual Hours on Job- Husband	0.000857∗∗∗	−0.00135∗∗∗
	(0.000105)	(7.04e-05)
Housing Status		
Rents	−1.241∗∗∗	−0.763∗∗∗
	(0.323)	(0.220)
Neither Rents nor Owns	−0.979	−0.720
	(0.809)	(0.500)
Income	−1.98e-07	−1.61e-07
	(1.45e-07)	(1.08e-07)
Wage Rate- Husband	−0.00201	−0.00570∗∗∗
	(0.00205)	(0.00118)
Wage Rate- Wife	−0.00531∗∗	−2.06e-05
	(0.00260)	(0.00146)
Observations	34,065	34,065

The marginal effects of the coefficient estimates from the fixed effects model with robust standard errors are presented in this table. ∗∗∗.

*p <* 0.01, ∗∗ *p <* 0.05, ∗ *p <* 0.1.

**Table A4:** Hours of Specific Unpaid Care Activities for Wives 2017–2019 Waves- Control Variable Results

	Caring for Child	Education	Caring for Adult	Personal Care	Shopping	Volunteering	Leisure
In the Past 30 Days, Everything is an effort							
Most of the time	1.043	0.406	−2.884	−2.454∗∗∗	−0.802	0.344	−1.412
	(4.878)	(0.616)	(2.360)	(0.883)	(0.732)	(0.463)	(1.800)
Some of the time	−0.112	0.243	−1.884	−2.769∗∗∗	−1.987∗∗∗	0.160	−1.730
	(4.628)	(0.650)	(2.035)	(0.830)	(0.663)	(0.420)	(1.642)
A little of the time	−1.484	0.253	−4.616∗∗	−3.444∗∗∗	−2.320∗∗∗	0.376	−0.578
	(4.644)	(0.670)	(2.272)	(0.851)	(0.701)	(0.429)	(1.679)
None of the time	−4.060	0.485	−4.246∗	−2.978∗∗∗	−2.165∗∗∗	0.0746	−1.737
	(4.716)	(0.671)	(2.177)	(0.866)	(0.676)	(0.412)	(1.673)
In the Past 30 Days, Nervous							
Most of the time	−12.04	0.641	−5.575∗	2.815∗∗	2.956∗∗∗	−0.220	2.825
	(9.016)	(1.036)	(2.923)	(1.145)	(0.921)	(0.762)	(2.076)
Some of the time	−2.638	0.230	−3.490	2.553∗∗	1.653∗∗	−0.860	2.750
	(7.734)	(0.978)	(2.305)	(1.064)	(0.751)	(0.763)	(1.871)
A little of the time	−2.289	0.0896	−3.268	3.244∗∗∗	2.056∗∗∗	−0.901	2.847
	(7.854)	(1.037)	(2.287)	(1.094)	(0.790)	(0.796)	(1.890)
None of the time	−4.654	0.393	−4.601∗∗	2.669∗∗	1.729∗∗	−0.685	2.191
	(7.772)	(1.023)	(2.260)	(1.098)	(0.774)	(0.791)	(1.882)
In the Past 30 Days, Sad							
Most of the time	7.747	1.594	8.474	−1.343	−2.352∗∗	1.218∗∗	−0.111
	(11.11)	(2.152)	(5.649)	(1.564)	(1.012)	(0.581)	(2.564)
Some of the time	2.448	2.569	10.89∗	−1.676	−1.614∗∗	0.922∗	−1.643
	(9.294)	(2.212)	(5.877)	(1.461)	(0.720)	(0.506)	(2.089)
A little of the time	−0.0104	2.230	14.11∗∗	−0.514	−1.552∗∗	0.883∗	0.805
	(9.670)	(2.304)	(6.220)	(1.561)	(0.782)	(0.521)	(2.200)
None of the time	0.767	2.107	12.89∗∗	−0.187	−1.262	0.866∗	0.277
	(9.731)	(2.327)	(6.389)	(1.562)	(0.770)	(0.519)	(2.143)
Number of People	4.678∗∗∗	−0.0975	0.868	0.204	0.541∗∗	−0.144	−0.0524
	(1.355)	(0.116)	(0.768)	(0.296)	(0.230)	(0.134)	(0.405)
Health Status- Head							
Very Good	−4.145∗∗	0.183	−0.257	0.0809	−0.168	0.473∗∗	0.431
	(1.709)	(0.267)	(0.868)	(0.428)	(0.256)	(0.220)	(0.501)
Good	−6.869∗∗∗	−0.0890	0.857	−0.231	−0.170	0.614∗∗∗	0.133
	(1.935)	(0.321)	(1.237)	(0.456)	(0.301)	(0.233)	(0.608)
Fair	−7.705∗∗∗	−0.406	4.293∗∗	−0.621	−1.242∗∗∗	0.155	−0.136
	(2.452)	(0.502)	(1.690)	(0.597)	(0.441)	(0.292)	(0.846)
Poor	−6.777∗∗	−0.561	19.29∗∗∗	1.368	−0.876	0.642	−2.643∗∗
	(3.038)	(0.582)	(4.426)	(0.971)	(0.613)	(0.559)	(1.323)
Health Status- Wife							
Very Good	1.414	0.202	1.145	−0.538	0.188	−0.240	−0.551
	(1.595)	(0.239)	(0.943)	(0.344)	(0.324)	(0.250)	(0.647)
Good	2.893	0.193	−1.223	−0.856∗∗	−0.161	−0.759∗∗	−1.267∗
	(1.849)	(0.311)	(1.314)	(0.412)	(0.366)	(0.328)	(0.756)
Fair	5.261∗∗	0.726∗∗	−0.669	−0.155	0.153	−0.312	−1.711∗
	(2.608)	(0.366)	(1.627)	(0.558)	(0.522)	(0.369)	(1.012)
Poor	6.696	0.277	−5.991∗∗	1.200	−0.374	−0.688∗	−0.224
	(4.155)	(0.392)	(2.594)	(0.871)	(0.722)	(0.383)	(1.321)
Both Smoke	−3.937	−0.724	−9.579∗∗	0.0835	−0.676	0.786	−2.409
	(4.963)	(0.936)	(3.767)	(1.120)	(1.062)	(0.612)	(2.151)
Wife Currently Smokes	−0.274	0.681	6.573∗∗	0.876	−0.150	−0.382	2.682∗
	(2.348)	(0.766)	(3.129)	(0.610)	(0.785)	(0.317)	(1.600)
Husband Currently Smokes	2.943	−0.0161	4.659∗	0.480	0.963	−0.390	2.851∗∗∗
	(3.605)	(0.612)	(2.394)	(0.930)	(0.764)	(0.449)	(1.083)
Age- Husband	−0.918∗	0.0306	0.0360	0.232	0.0413	0.117	0.0453
	(0.498)	(0.108)	(0.366)	(0.147)	(0.111)	(0.0719)	(0.212)
Age- Wife	0.335	−0.0665	0.103	−0.165	0.0261	−0.0934	0.123
	(0.577)	(0.111)	(0.253)	(0.146)	(0.114)	(0.0694)	(0.194)
Number of Children	5.057∗∗	−0.156	0.195	0.0743	−0.0898	0.158	−0.208
	(2.102)	(0.198)	(1.151)	(0.351)	(0.260)	(0.149)	(0.475)
Received College Degree- Wife							
Yes	6.421	−0.585	1.206	1.166	1.361	0.963	−1.190
	(17.36)	(2.153)	(3.840)	(1.638)	(1.753)	(1.431)	(4.127)
No	17.73	4.985	0.475	1.453	2.352	−0.0991	−5.811
	(19.38)	(3.234)	(3.964)	(1.706)	(1.678)	(1.656)	(4.936)
Received College Degree- Husband							
Yes	−14.47	1.961	4.303	1.443	0.456	−0.584	6.291
	(13.36)	(1.602)	(3.090)	(1.500)	(1.936)	(0.792)	(4.441)
No	−13.88	2.109	3.458	1.553	−0.351	−0.209	3.513
	(12.22)	(2.068)	(2.657)	(1.218)	(1.075)	(0.852)	(4.567)
Annual Hours on Job- Wife	−0.00288∗∗∗	−0.000516∗∗∗	−0.000208	−0.000136	−0.000624∗∗∗	−0.000442∗∗∗	−0.00154∗∗∗
	(0.000887)	(0.000178)	(0.000450)	(0.000229)	(0.000152)	(0.000159)	(0.000336)
Annual Hours on Job- Husband	−0.000158	0.000128	0.000102	0.000516∗∗	−1.38e-05	−0.000378∗∗∗	−0.000625∗∗
	(0.000940)	(0.000157)	(0.000222)	(0.000227)	(0.000142)	(0.000124)	(0.000304)
Housing Status							
Rents	−0.668	0.795	0.456	−0.192	−0.801∗	−0.213	1.350∗
	(3.068)	(0.486)	(0.957)	(0.435)	(0.436)	(0.224)	(0.722)
Neither Rents nor Owns	−5.519	1.947	14.07∗∗∗	−0.749	−0.918	−0.623	0.745
	(8.125)	(1.344)	(5.168)	(1.180)	(0.907)	(0.581)	(1.575)
Income	2.74e-06	−2.09e-08	−6.91e-08	1.92e-07∗	−1.78e-07	−2.06e-07∗∗	−4.53e-07
	(2.01e-06)	(3.90e-08)	(7.19e-08)	(1.06e-07)	(1.35e-07)	(9.63e-08)	(3.25e-07)
Wage Rate- Husband	−0.0201	8.11e-06	0.00294	0.00527	0.00484	−0.000719	−0.000909
	(0.0131)	(0.00268)	(0.00421)	(0.00399)	(0.00297)	(0.00171)	(0.00609)
Wage Rate- Wife	−0.00302	−0.00322∗∗∗	0.000120	0.00467∗∗	0.00198	−0.000871	0.00322
	(0.00559)	(0.00111)	(0.00429)	(0.00219)	(0.00216)	(0.000978)	(0.00335)

Religion dummy variables were omitted for length. Please email the authors for those results. ∗∗∗ *p <* 0.01, ∗∗ *p <* 0.05, ∗ *p <* 0.1.

**Table A5:** Hours of Specific Unpaid Care Activities for Husbands 2017–2019 Waves- Control Variable Results

	Caring for Child	Education	Caring for Adult	Personal Care	Shopping	Volunteering	Leisure
In the Past 30 Days, Everything an effort							
Most of the time	4.633	−0.439	0.267	−0.742	0.452	−1.101∗∗∗	−4.522∗∗
	(4.088)	(0.549)	(1.193)	(0.931)	(0.441)	(0.427)	(2.245)
Some of the time	−0.970	−0.404	−0.217	−2.095∗∗	−0.403	−0.673	−5.268∗∗
	(3.737)	(0.603)	(0.817)	(0.825)	(0.437)	(0.477)	(2.213)
A little of the time	−0.301	−0.348	−0.727	−1.918∗∗	−0.0189	−0.556	−5.450∗∗
	(3.670)	(0.619)	(0.887)	(0.863)	(0.450)	(0.469)	(2.301)
None of the time	−0.211	−0.331	−0.707	−2.109∗∗	−0.0383	−0.521	−4.665∗∗
	(3.686)	(0.597)	(0.804)	(0.884)	(0.451)	(0.466)	(2.207)
In the Past 30 Days, Nervous							
Most of the time	5.305	−1.294∗∗	1.015	−0.190	0.108	0.324	−0.335
	(4.989)	(0.568)	(1.892)	(0.866)	(0.673)	(0.355)	(3.186)
Some of the time	8.197	−0.870	−1.162	0.174	0.107	0.0486	0.689
	(5.164)	(0.599)	(0.854)	(0.811)	(0.537)	(0.372)	(3.174)
A little if the time	6.273	−1.191∗	−0.356	1.044	0.156	−0.0245	1.712
	(5.071)	(0.618)	(0.836)	(0.830)	(0.569)	(0.395)	(3.211)
None of the time	7.416	−0.900	−0.694	0.595	0.263	0.213	1.276
	(5.055)	(0.631)	(0.861)	(0.829)	(0.569)	(0.393)	(3.214)
In the past 30 days, sad							
Most of the time	−1.674	0.947∗	−0.374	−2.789∗∗	−0.620	−1.186	−9.180∗∗
	(5.039)	(0.506)	(1.180)	(1.282)	(0.545)	(0.912)	(4.086)
Some of the time	1.167	1.465∗∗∗	−0.141	−0.261	−1.062∗∗	−0.974	−6.629
	(5.239)	(0.497)	(0.913)	(0.985)	(0.476)	(0.975)	(4.058)
A little of the time	−0.624	1.532∗∗∗	0.534	−0.118	−1.086∗∗	−1.093	−5.806
	(5.292)	(0.525)	(1.312)	(1.060)	(0.511)	(0.956)	(4.285)
None of the time	−0.768	1.295∗∗	0.585	−0.0210	−0.724	−1.280	−6.285
	(5.359)	(0.512)	(1.273)	(1.015)	(0.495)	(0.949)	(4.224)
Number of People	3.587∗∗∗	−0.00357	−0.611	−0.0813	−0.0855	0.0685	0.766
	(1.181)	(0.161)	(0.490)	(0.192)	(0.130)	(0.0747)	(0.557)
Health Status- Husband							
Very Good	0.176	0.161	0.639	−0.0952	−0.224	−0.0754	0.0869
	(1.373)	(0.165)	(0.569)	(0.245)	(0.180)	(0.187)	(0.616)
Good	−0.424	0.242	0.120	−0.0288	−0.441∗	0.231	−0.758
	(1.596)	(0.196)	(0.582)	(0.300)	(0.251)	(0.201)	(0.737)
Fair	−2.313	0.549∗	1.401∗	−0.619	−0.818∗∗	0.129	−1.200
	(1.946)	(0.293)	(0.820)	(0.442)	(0.329)	(0.242)	(1.143)
Poor	−0.776	−0.113	0.974	−0.501	−0.597∗	0.128	−1.589
	(2.185)	(0.357)	(0.793)	(0.713)	(0.356)	(0.255)	(3.026)
Health Status- Wife							
Very Good	−0.106	0.0569	−0.111	0.174	0.00655	0.0326	−1.479∗
	(1.131)	(0.282)	(0.247)	(0.253)	(0.222)	(0.196)	(0.848)
Good	1.432	−0.166	0.0647	0.444	0.0844	−0.229	−1.363
	(1.346)	(0.288)	(0.323)	(0.307)	(0.229)	(0.204)	(1.031)
Fair	2.565	0.212	−0.287	0.587	0.300	−0.536∗∗	−0.215
	(1.815)	(0.344)	(0.734)	(0.462)	(0.283)	(0.226)	(1.404)
Poor	4.698	0.0800	1.936	1.031	−0.125	−0.0352	1.199
	(3.397)	(0.492)	(2.441)	(0.647)	(0.373)	(0.257)	(1.775)
Both Smoke	6.299	−1.014∗∗∗	−3.821∗	−1.082	−1.119∗∗	0.238	1.433
	(4.334)	(0.388)	(2.191)	(1.073)	(0.483)	(0.323)	(2.442)
Wife Currently Smokes	1.333	−0.0287	0.0547	1.857∗∗∗	0.237	−0.568∗	1.058
	(1.573)	(0.228)	(0.616)	(0.538)	(0.282)	(0.303)	(1.666)
Husband Currently Smokes	−0.104	1.185∗∗∗	1.781∗	1.071∗	0.912∗∗	0.0986	0.862
	(3.169)	(0.411)	(1.056)	(0.590)	(0.400)	(0.259)	(1.310)
Age- Husband	−2.007∗	−0.356∗	−0.0330	−0.00869	−0.0516	0.0698∗	−0.487
	(1.028)	(0.215)	(0.0796)	(0.102)	(0.0671)	(0.0414)	(0.364)
Age- Wife	1.631	0.160	0.0299	0.118	0.0481	−0.0449	0.649∗
	(1.054)	(0.135)	(0.0877)	(0.0941)	(0.0792)	(0.0339)	(0.360)
Number of Children	0.0308	−0.409∗	0.222	0.295	0.0665	0.191∗	1.287∗∗
	(1.455)	(0.237)	(0.390)	(0.254)	(0.163)	(0.0999)	(0.585)
Received College Degree- Wife							
Yes	−1.061	−3.812∗	−1.399	2.409	−0.488	0.463	−4.410
	(6.508)	(2.220)	(1.540)	(2.139)	(0.962)	(1.138)	(5.991)
No	−1.262	−3.101	−0.935	2.553	−0.538	−0.348	−7.899
	(7.066)	(2.279)	(1.577)	(2.126)	(0.892)	(1.246)	(5.502)
Received College Degree- Husband							
Yes	2.756	1.227	−0.0655	−2.674	0.731	−0.272	3.283
	(5.070)	(3.106)	(1.129)	(2.669)	(0.833)	(0.704)	(4.231)
No	−2.508	1.756	−0.0377	−0.491	0.803	−0.340	1.204
	(4.760)	(2.633)	(0.793)	(1.753)	(0.658)	(0.782)	(3.543)
Annual Hours on Job- Wife	−0.000239	−5.49e-05	0.000449∗∗	−0.000240	−0.000159	−0.000160∗	−0.000783∗
	(0.000652)	(9.32e-05)	(0.000210)	(0.000163)	(0.000103)	(9.14e-05)	(0.000421)
Annual Hours on Job- Husband	−0.00290∗∗∗	−0.000163	2.74e-05	0.000277∗	4.46e-05	−0.000122	−0.00196∗∗∗
	(0.000717)	(0.000100)	(0.000132)	(0.000163)	(9.31e-05)	(8.51e-05)	(0.000374)
Housing Status							
Rents	−2.204	0.0171	−1.882∗∗	0.264	−0.211	−0.222	0.313
	(2.343)	(0.355)	(0.895)	(0.383)	(0.304)	(0.145)	(1.325)
Neither Rents Nor Owns	−7.465	0.532	10.52∗∗	1.082	−0.378	−0.547	1.865
	(6.661)	(0.550)	(4.224)	(0.850)	(0.610)	(0.366)	(2.037)
Income	5.83e-07∗∗	1.48e-08	−1.01e-07∗∗∗	7.08e-08	8.03e-08∗∗	5.18e-08	2.50e-07∗∗∗
	(2.39e-07)	(4.60e-08)	(3.56e-08)	(1.15e-07)	(3.93e-08)	(6.56e-08)	(8.89e-08)
Wage Rate- Husband	−0.0100	−0.00304∗	−0.00425∗	−0.000572	0.00399∗∗	0.00207	−0.0112
	(0.00839)	(0.00176)	(0.00228)	(0.00259)	(0.00199)	(0.00204)	(0.00796)
Wage Rate- Wife	0.00660	−0.00169	0.000556	−0.00147	−0.00362∗∗∗	0.00178∗∗	0.0119∗∗
	(0.00470)	(0.00124)	(0.000892)	(0.00148)	(0.00131)	(0.000862)	(0.00546)

Religion dummy variables were omitted for length. Please email the authors for results. ∗∗∗ *p <* 0.01, ∗∗ *p <* 0.05, ∗ *p <* 0.1.

**Table A6:** Cross Lagged Panel Using ML-SEM: Hours of Housework 2005–2019 Waves in AAFs versus Non AAFs

	Housework- Wife	Housework- Husband
**Alcohol Consumption Variables- Husband**		
Consumes Alcohol	1.324∗∗∗	0.735∗∗∗
	(0.390)	(0.257)
Heavy Alcohol Use	1.574∗	−0.543
	(0.849)	(0.441)
Number of Drinks	−0.0444	−0.00611
	(0.0657)	(0.0409)
Alcohol Frequency		
About Once a Month	−0.360	−0.135
	(0.316)	(0.221)
Several Times a Month	−0.809∗∗	0.00374
	(0.355)	(0.255)
Once a Week	−1.040∗∗∗	−0.405∗
	(0.368)	(0.244)
Several Times a Week	−0.709∗	−0.389
	(0.390)	(0.273)
Every Day	−1.041∗∗	−0.0202
	(0.520)	(0.363)
**Alcohol Consumption Variables- Wife**		
Consumes Alcohol	1.528∗∗∗	0.0359
	(0.415)	(0.294)
Heavy Alcohol Use	−1.174	0.373
	(1.099)	(1.667)
Number of Drinks	−0.122	−0.137∗∗
	(0.0859)	(0.0612)
Alcohol Frequency		
About Once a Month	−0.294	−0.227
	(0.282)	(0.198)
Several Times a Month	−0.437	−0.321
	(0.338)	(0.228)
Once a Week	0.221	−0.209
	(0.354)	(0.230)
Several Times a Week	−0.0765	−0.346
	(0.395)	(0.281)
Every Day	0.420	−0.682∗
	(0.560)	(0.396)
**Interaction Terms**		
Both Spouses Consume Alcohol	1.128∗∗∗	−1.607∗∗∗
	(0.415)	(0.293)
Heavy Alcohol Use- Both Spouses	−1.499	−2.296
	(1.623)	(1.737)

The total effects of the coefficient estimates from the SEM model with robust standard errors are presented in this table. The control variables in the regression include: the number of people, the wife smokes cigarettes, the husband smokes cigarettes, interaction term between the two, age of husband and wife, the hours worked by both the husband and wife, religion, housing status, income of the household, the hourly earnings of the husband, and the hourly earnings of the wife. To avoid over-specification of the model, only one category of health status is included for the husband and wife. ∗∗∗ *p <* 0.01, ∗∗ *p <* 0.05, ∗ *p <* 0.1.

**Table A7:** Dynamic Panel Data Models using the Generalized Method of Mo- ments (GMM) Framework: Hours of Housework 2005–2019 Waves in AAFs ver-sus Non AAFs

	Housework- Wife	Housework- Husband
**Alcohol Consumption Variables- Husband**		
Consumes Alcohol	−14.14	5.850∗∗∗
	(9.550)	(2.268)
Heavy Alcohol Use	4.696∗	0.475
	(2.524)	(1.246)
Number of Drinks	−0.801	−0.261∗∗
	(0.734)	(0.125)
Alcohol Frequency		
About Once a Month	−5.939	−0.978
	(5.910)	(0.663)
Several Times a Month	0.262	0.0881
	(7.323)	(1.097)
Once a Week	−7.146	−0.278
	(5.790)	(0.926)
Several Times a Week	−4.689	−0.207
	(6.065)	(0.990)
Every Day	−5.383	0.401
	(7.981)	(1.487)
**Alcohol Consumption Variables- Wife**		
Consumes Alcohol	−6.975	19.10∗∗∗
	(5.915)	(7.248)
Heavy Alcohol Use	−7.152∗	−1.497
	(4.276)	(4.427)
Number of Drinks	−0.304	0.811
	(0.271)	(0.786)
Alcohol Frequency		
About Once a Month	−0.281	5.686∗∗
	(0.977)	(2.744)
Several Times a Month	−0.885	−2.250
	(1.554)	(3.662)
Once a Week	0.884	−2.926
	(1.596)	(4.019)
Several Times a Week	0.242	−3.326
	(1.827)	(3.549)
Every Day	0.288	−4.320
	(2.706)	(5.135)
**Interaction Terms**		
Both Spouses Consume Alcohol	14.64	−20.18∗∗∗
	(9.304)	(7.505)
Heavy Alcohol Use- Both Spouses	4.229	1.401
	(11.53)	(9.722)

The coefficient estimates from the xtabond2 command in STATA with robust standard errors are presented in this table. ∗∗∗ *p <* 0.01, ∗∗ *p <* 0.05, ∗ *p <* 0.1.

## Data Availability

Data will be made available on request.

## Footnotes

1 The Hausman test yielded a p-value of 0 and a chi-squared statistic of 148.72, indicating that the fixed effects model was appropriate.
